# Establishment of an artificial urine model in vitro and rat or pig model in vivo to evaluate urinary crystal adherence

**DOI:** 10.1038/s41598-024-62766-w

**Published:** 2024-05-25

**Authors:** Kana Hayashi, Katsumi Shigemura, Hiroshi Tanimoto, Kazuo Kumagai, Ralph Rolly Gonzales, Young-Min Yang, Koki Maeda, Hideto Matsuyama, Masato Fujisawa

**Affiliations:** 1 Division of Infectious Diseases, Department of Public Health, Kobe University Graduate School of Health Sciences, 7-10-2 Tomogaoka Suma-Ku, Kobe, 654-0142 Japan; 2 Department of Urology, Teikyo University Graduate School of Medicine, 2-11-1 Kaga, Itabashi-Ku, Tokyo, 173-8605 Japan; 3 Research Center for Membrane and Film Technology, Kobe University, 1-1 Rokkodaicho, Nada-Ku, Kobe, 657-8501 Japan; 4 Department of Chemical Science and Engineering, Kobe University, 1-1 Rokkodaicho, Nada-Ku, Kobe, 657-8501 Japan; 5 Division of Urology, Kobe University Graduate School of Medicine, 7-5-1 Kusunoki-Cho, Chuo-Ku, Kobe, 650-0017 Japan

**Keywords:** Biochemistry, Biotechnology, Urology

## Abstract

The current study aimed to establish an experimental model in vitro and in vivo of urinary crystal deposition on the surface of ureteral stents, to evaluate the ability to prevent crystal adhesion. Non-treated ureteral stents were placed in artificial urine under various conditions in vitro. In vivo, ethylene glycol and hydroxyproline were administered orally to rats and pigs, and urinary crystals and urinary Ca were investigated by Inductively Coupled Plasma-Optical Emission Spectrometer. in vitro, during the 3- and 4-week immersion periods, more crystals adhered to the ureteral stent in artificial urine model 1 than the other artificial urine models (p < 0.01). Comparing the presence or absence of urea in the composition of the artificial urine, the artificial urine without urea showed less variability in pH change and more crystal adhesion (p < 0.05). Starting the experiment at pH 6.3 resulted in the highest amount of crystal adhesion to the ureteral stent (p < 0.05). In vivo, urinary crystals and urinary Ca increased in rat and pig experimental models. This experimental model in vitro and in vivo can be used to evaluate the ability to prevent crystal adhesion and deposition in the development of new ureteral stents to reduce ureteral stent-related side effects in patients.

## Introduction

Urological patients, especially those with ureteral stricture or ureteral stones due to ureteral cancer, are at high risk of urinary tract obstruction or urinary flow stagnation, leading to renal insufficiency. Usually, a ureteral stent is placed to secure the urinary passage and prevent this. However, if a ureteral stent is left in place for a long period, it may become obstructed by the adhesion of foreign matter or crystal formation, which itself causes urinary tract obstruction^1,2^.

There are two main mechanisms of urinary crystal adhesion to ureteral stents, one mediated by bacteria and one where urinary crystal components are deposited directly on the surface of the stent without bacteria. With the bacterial mechanism (1), proteins in the urine are adsorbed to the surface of the stent and bacteria in the urine adhere and proliferate using this protein as a scaffold to form biofilm. Some urinary bacteria produce urease, which breaks down urea to produce ammonia. The ammonia raises the pH, causing calcium (Ca) dissolved in the urine to precipitate and form crystals. In the direct deposit mechanism (2), the urinary Ca concentration increases because of excessive intake of calcium oxalate, and the Ca salt precipitates directly onto the surface of the ureteral stent without bacterial intervention. Inhibition of either mechanism can prevent urinary crystal adhesion.

Various attempts have been made to increase the durability and prevent the obstruction of ureteral stents; for instance, hydrogel coating. In this method, a hydrophilic coating is applied to the surface of the ureteral stent to prevent urinary crystals and protein from adhering^3^. In addition, there are Teflon coatings that have heat resistance, durability, and smoothness, and smooth coatings to reduce the vulnerable surface area^4^. There two main types of coating that inhibit the adhesion of bacteria to stents are silver sulfadiazine and antibacterial agents^5–7^. There also are reports from Japan examining the clinical effects of changing the shape of the stent to a loop and using metal^8,9^.

To evaluate the ability of new ureteral stent designs and materials to resist obstruction, in vitro experiments using artificial urine and in vivo experiments using animals such as rats, pigs, monkeys have been performed. However, the experimental protocols and evaluation criteria vary, and no experimental method has been established that focuses on urinary crystal adhesion to ureteral stents. Therefore, in this study we established new experimental models using artificial urine, rat, and pig to evaluate the ability of ureteral stent to resist urinary crystal adhesion, which may prove useful for testing new ureteral stents in the future.

## Materials and methods

### Materials

In this study, non-coating ureteral stents made of ethylene–vinyl acetate copolymer (Percuflex Urinary Diversion Stent; Boston Scientific, USA) were used. The ureteral stents were cut to 1.0 cm for artificial urine and rat experiments, and 26.0 cm for pig experiments.

### Artificial urine experimental model

We conducted the artificial urine experiment in the order “Artificial urine experimental model”, “Evaluation of the need for urea”, and “Evaluation of the most appropriate pH conditions” (see Supplementary Fig. S1 online).

#### Comparison of amounts of crystal adhesion by artificial urine composition

To investigate crystal adhesion to the surface of the ureteral stents, three types of artificial urine with different compositions were used with reference to previous studies^10–12^. The composition of each artificial urine is shown in Table 1. All components were individually 0.22 μm filter sterilized with a Millex Syringe-driven Filter Unit (Merck Millipore Ltd., Ireland), and mixed in order as shown in Table 1. The artificial urine pH was adjusted to 6.00 with hydrochloric acid and sodium hydroxide.

**Table 1:** The composition of the three artificial urine experimental models.

Component	MW	Model 1 (mM)	Model 2 (mM)	Model 3 (mM)
NaCl	58.44	105.50	78.71	30.05
NaH_2_PO_4_	119.98	32.30		18.67
Na_2_HPO_4_	141.96			4.67
KH_2_PO_4_	136.09		20.58	
Na_3_C_6_H_5_O_7_	258.06	3.21	2.52	2.45
MgSO_4_·7H_2_O	246.47	3.85		4.39
Na_2_SO_4_	142.04		16.19	11.97
MgCl_2_·6H_2_O	203.31		3.20	
KCl	74.55	63.70	21.46	
CaCl_2_	110.98	5.75	4.42	1.66
Na_2_C_2_O_4_	134.00	0.32	0.15	0.19
NH_4_Cl	53.49	27.60	18.70	23.67
CO (NH_2_)_2_	60.06	166.50	416.25	249.75
C_5_H_4_N_4_O_3_	168.11			1.49
C_4_H_7_N_3_O	113.12			7.79
pH		6.00	6.00	6.00

Table shows the composition of the three artificial urine experimental models. The pH of artificial urine was adjusted to pH 6.00 with hydrochloric acid and sodium hydroxide.

The ureteral stents (n = 5, respectively) were sterilized and soaked in each artificial urine at 37C, and taken out after 4 weeks. After washing with MQ water and drying, the surfaces were observed by stereo microscope (Stemi 305 Cam; Carl Zeiss Microscopy GmbH, Germany). The number of crystals adhering to the ureteral stent in a 2 mm × 2 mm area under the stereo microscope was counted and analyzed.

#### Evaluation of the need for urea in artificial urine model 1

To investigate whether urea affects crystal adhesion to the ureteral stent, the amount of crystal adhesion was compared with and without urea in the artificial urine. The two types of artificial urine in model 1 were prepared as described above. The ureteral stents (n = 5, respectively) were sterilized and soaked in each artificial urine at 37 °C, and taken out after 4 weeks and 5 weeks. After washing with MQ water and drying, surfaces were observed by stereo microscope. The number of crystals adhering to the ureteral stent in a 2 mm × 2 mm area under the stereo microscope was counted and analyzed.

#### Evaluation of the most appropriate pH conditions

To evaluate the most appropriate pH conditions for crystal adherence to the surface of the ureteral stent, adhesion was compared between artificial urines with different pH. Four types of artificial urine model 1, with pH 6.00, pH 6.10, pH 6.20, and pH 6.30, were prepared as described above. The ureteral stents (n = 5, respectively) were sterilized and soaked in each artificial urine at 37C, and taken out after 4 weeks. After washing with MQ water and drying, the surfaces were observed by stereo microscope. The number of crystals adhering to the ureteral stent in a 2 mm × 2 mm area under the stereo microscope was counted and analyzed.

Quantitative analysis of crystal (Ca, magnesium (Mg) and phosphorus (P)) adherence to the stents was performed by inductively coupled plasma—optical emission spectrometry (ICP-OES, ICPE9800, Shimadzu, Japan). The stents were first placed in 1 mL 8% HNO_3_ and placed in ultrasonication bath to dissolve the crystals. The solution was afterwards diluted using 1% HNO_3_ (aq) prior to ICP-OES analysis^13^. The surface morphology and elemental mapping of the stents were characterized using a scanning electron microscope (SEM, Phenom ProX, Thermo Fisher Scientific, Japan) and SEM-coupled with energy dispersive spectroscopy (SEM–EDS) analysis. The stent samples were freeze-dried and sputter-coated with platinum prior to imaging.

On the determination of oxalic acid concentrations, each stent (1 cm) was washed three times with ultrapure water and added with 1 ml of 0.1% nitric acid and kept for 24 h at room temperature to dissolve stones attached on the surface. The concentration of oxalic acid was determined by an LC–MS/MS system (LCMS-8045, Shimadzu, Japan) equipped with a Tosoh TSKgel ODS-120H column (2 mm i.d. × 50 mm, 1.9 μm) using 0.1% formic acid in ultrapure water as a mobile phase at a flow rate of 0.2 ml/min with a tandem-mass (MS/MS) detector.

### Rat experimental model

To promote formation of urinary crystal, water containing 0.4% ethylene glycol and 1% ammonium chloride was orally administered to rats (n = 4) as drinking water on day 1 ~ day 14, day 25 ~ day 28, and day32 ~ day 35^14^. The ureteral stent was placed in the bladder of these calculus model rats on day 14. We administered 20 mg/kg (body weight) of antibiotics (ceftriaxone) intraperitoneally on the day of stenting to prevent infection. Four weeks later, each ureteral stent was removed from the bladder, washed with MQ water, dried, and then subjected to surface observation with a stereo microscope. We collected urine once a week after starting the water to promote formation of urinary crystals. After collecting the urine and preparing a urine sediment specimen, urinary crystals on days 7, 28, 35 and 42 were examined under a microscope (BZ-X710; KEYENCE, Osaka, Japan) at 200 × magnification to determine changes over time. During the experimental period, general condition observation, weight measurement, and urine collection were performed to evaluate the safety of this experiment.

During surgery, isoflurane was inhaled at a 5% concentration and maintained at 2–3%. We euthanized rats due to cervical dislocation.

### Pig experimental model

The pigs were fed a high-fiber diet to minimize weight changes, as large changes in body weight could affect the test plan^18,19^. To promote formation of urinary crystals, 5% hydroxyproline-added feeds were orally administered to pig on all days. 1% ethylene glycol and vitamin D-added feeds were orally administered to pig on day 1 ~ day 19, day 26 ~ day 33 and day 57 ~ 71^17–20^. The ureteral stents were placed in the left and right ureters of these pigs for 4 weeks. we administered 1 g/head of antibiotic (cephazolin sodium for intramuscular injection) for 3 days from the date of stent placement to prevent infection. After four weeks, the ureteral stents were removed from the ureters, washed with MQ water, dried, and then subjected to surface observation by stereo microscope with quantitative analysis of crystal (Ca, Mg, P) adherence to the stents performed by ICP-OES at three sites: kidney, ureter and bladder. In addition, we collected urine twice a week. 5 views were observed under a microscope (Axiovert 200-30; Carl Zeiss Microscopy GmbH, Germany) at 100 × magnification (Low Power Field), and the average value was calculated by counting the crystals in the urine.

During surgery, a mixture of intramuscular ketamine hydrochloride (10 mg/kg), xylazine hydrochloride (2 mg/kg) and atropine sulphate injection (0.5 mg/body) was administered intramuscularly and sedated. After sedation, isoflurane was inhaled at a 5% concentration and maintained at 2–3%. We cut the aorta of the pig under deep anesthesia, released blood and ensured that arterial oxygen saturation was '0' and euthanized.

### Ethical approval

This study of rat was approved by Japanese Association for Laboratory Animal Science (JALAS) (Permission number P200504) and carried out according to the Kobe University Animal Experimentation Regulations. This study for pig approved by JALAS (Permission number IVT22-166) and carried out according to IBTEC Corporation Animal Experimentation Regulations. All procedures in the animal studies were performed in accordance with the institutional ethical standards in compliance with the ARRIVE guidelines (https://arriveguidelines.org) and all relevant guidelines and regulations. This article does not contain any studies with human participants performed by any of the authors.

### Statistical analysis

Comparisons between two different groups were performed using Student’s T-test. Comparisons between the three different groups were analyzed by one-way ANOVA, and Tukey's multiple comparisons were performed for those that showed differences. Statistical differences among means were considered significant when* p* < 0.05.

## Results

### Artificial urine experimental model

#### Comparison of amount of crystal adhesion by artificial urine composition

The pH of artificial urine model 1 was 6.06 after 1 week and 6.74 after 4 weeks. The pH of artificial urine model 2 was 6.54 after 1 week and 6.54 after 4 weeks. The pH of artificial urine model 3 was 5.98 after 1 week and 6.74 after 4 weeks.

**Figure 1:**
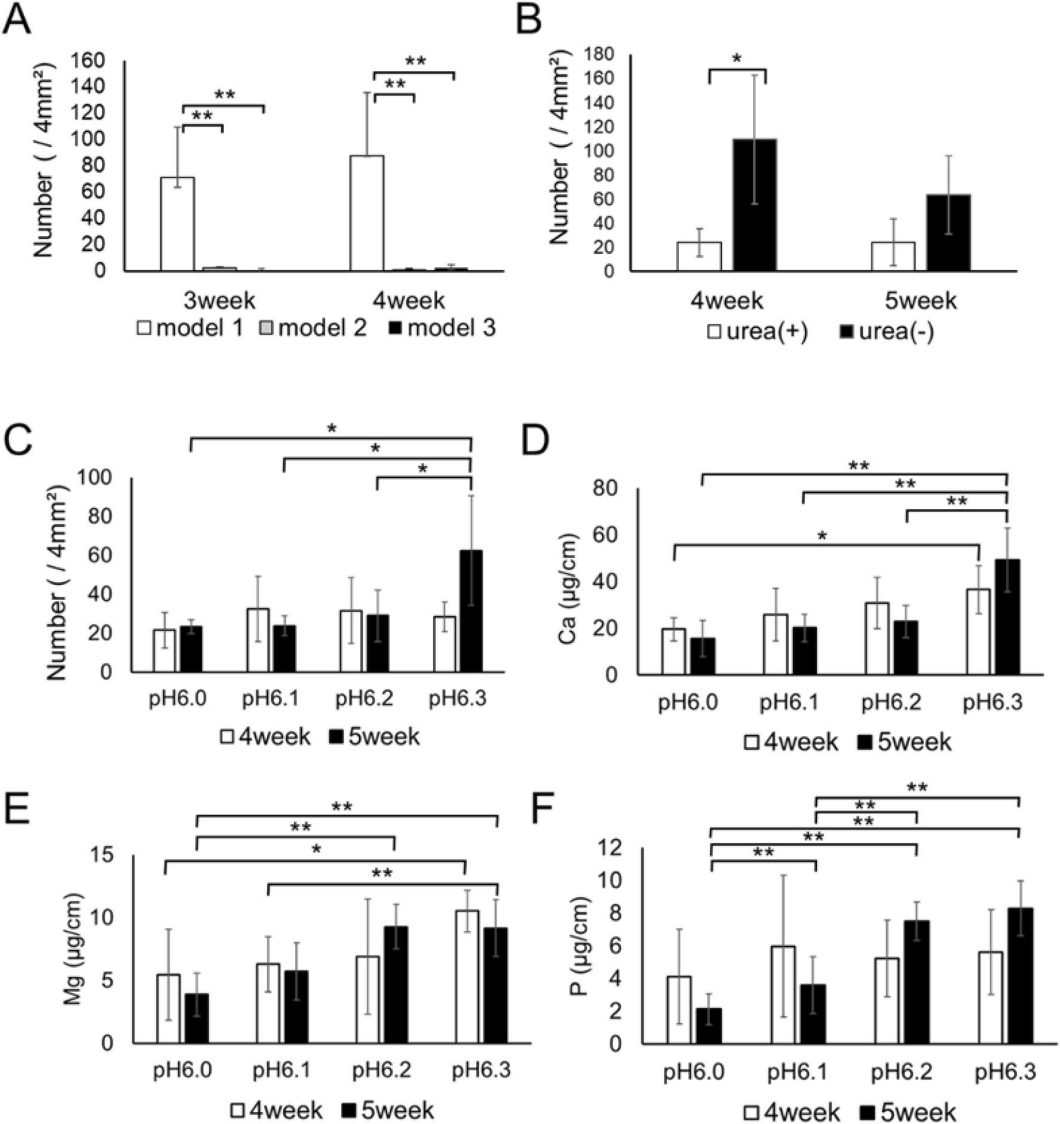
The number of crystals attached to ureteral stents immersed in artificial urine models under various conditions. (**A**) shows the number of crystals attached to ureteral stents immersed in three artificial urine models after 3 and 4 weeks of immersion (n = 5, average $\pm$ SD bars, **p < 0.01). (**B**) shows the number of crystals attached to ureteral stents immersed in artificial urine with and without urea after 4 and 5 weeks of immersion (n = 5, average ± SD bars, *p < 0.05). (**C**) The number of crystals, (D) Ca, (E) Mg, and (F) P attached to ureteral stents immersed in artificial urine with different pH conditions after 4 and 5 weeks of immersion (n = 5, average ± SD bars, *p < 0.05, **p < 0.01).

At the 3-week immersion point, the number of crystals on the ureteral stent immersed in artificial urine model1 was higher than in artificial urine model 2 and 3 (*p* = 0.00264, *p* = 0.00216, respectively). After the 4-week immersion period, the number of crystals on the ureteral stent immersed in artificial urine model1 was higher than in artificial urine model 2 and 3 (*p* = 0.00202, *p* = 0.00200, respectively) (Fig. 1A).

#### Evaluation of the need for urea in artificial urine model 1

The pH of the artificial urine containing urea was 6.00 after 1 week and 7.98 after 5 weeks. The pH of artificial urine without urea was 6.10 after 1 week and 6.30 after 5 weeks.

At the 4-week immersion period, the number of crystals on the ureteral stent immersed in urea-free artificial urine was higher than for urea-containing artificial urine (*p* = 0.0140). At the 5-week immersion period, the number of crystals on ureteral stents immersed in urea-free artificial urine tended to be higher than in urea-containing artificial urine, although there was no predominant difference (Fig. 1B).

#### Evaluation of the most appropriate pH condition

The artificial urine with a starting pH of pH 6.00 was still 6.00 after 1 week and 6.00 after 5 weeks. The pH of artificial urine with a starting pH of 6.10 was 6.00 after 1 week and 6.14 after 5 weeks. The pH of artificial urine with a starting pH of 6.20 was 6.20 after 1 week and 6.28 after 5 weeks. The pH of artificial urine with a starting pH of 6.30 was 6.30 after 1 week and 6.56 after 5 weeks. At the 5-week immersion period, the number of crystals on the ureteral stent immersed in artificial urine with a starting pH of pH 6.30 was higher than for artificial urine with a starting pH of pH6.00, pH6.10, and pH6.20 (*p* = 0.0143, *p* = 0.0154, *p* = 0.0387, respectively) (Fig. 1C).

Regarding the quantitative analysis of Ca, Mg and P at the 4-week immersion period, the amount of Ca and Mg on the ureteral stent immersed in artificial urine with a starting pH of pH 6.30 was higher than in artificial urine with a starting pH of pH6.00 (*p* = 0.0258,* p* = 0.0135, respectively) (Fig. 1D,E). At the 5-week immersion period, the amount of Ca, Mg and P on the ureteral stent immersed in artificial urine with a starting pH of pH 6.30 was higher than in artificial urine with a starting pH of pH6.00 (*p* < 0.01, respectively) (Fig. 1D,E,F).

The SEM observation confirmed the presence of crystals on the surface of the ureteral stent immersed in artificial urine, and C, O, Ca, and P were detected by SEM–EDS analysis (see Supplementary Fig. S2 online). The concentration of oxalic acid on the stent was 8.67 µg/cm stent as determined by LC–MS/MS.

### Rat experimental model

**Figure 2:**
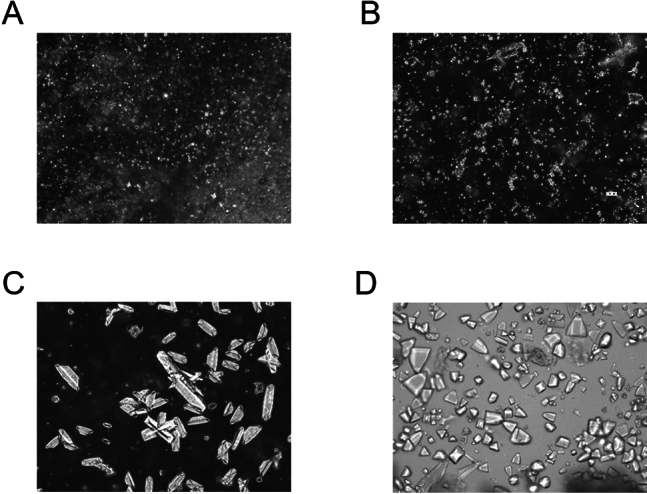
Changes over time in urinary crystals in rats. (**A**) Urinary crystals on day 7, (**B**) Urinary crystals on day 28, (**C**) Urinary crystals on day 35. (**D**) Urinary crystals on day 42. Magnification is 200x.

There was a tendency for body weight to decrease during the period when 0.4% ethylene glycol and 1% ammonium chloride was administered, but overall, body weight was increasing. No noticeable crystals were found in the urine on days 7 and 28, but on days 35 and 42, noticeable crystals were observed. The shape of the crystals was an elongated octahedral structure on day 35 and a regular octahedral structure on day 42 (Fig. 2).

### Pig experimental model

**Figure 3:**
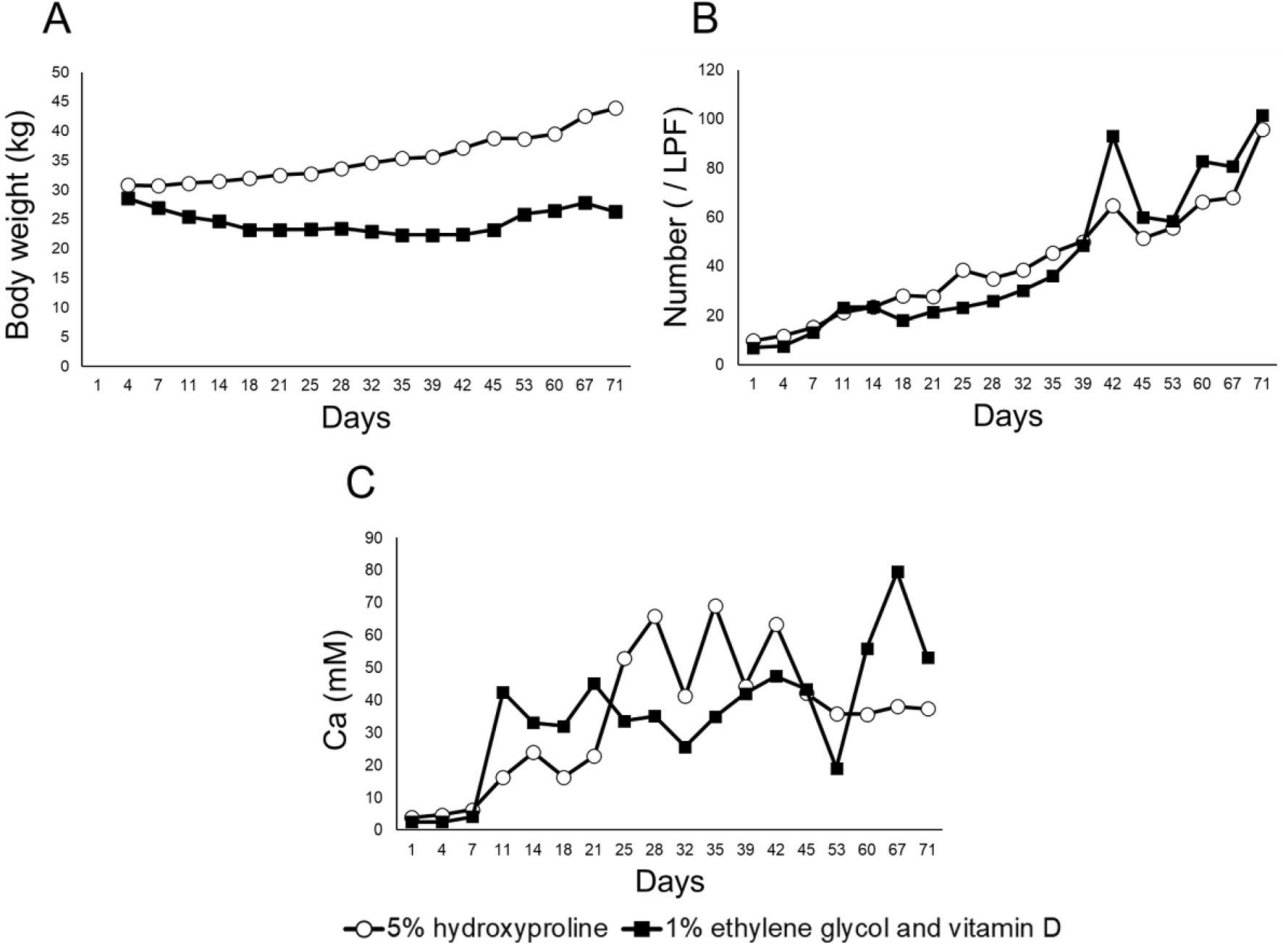
Body weight and urinary crystals in pigs. (**A**) Body weight, (**B**) The average number of urinary crystals, (**C**) Ca in urine in pigs fed 5% hydroxyproline and 1% ethylene glycol and vitamin D. Period of 1% ethylene glycol and vitamin D fed: day 1 ~ day 19, day 26 ~ day 33, day 57 ~ 71. On day 45, the ureteral stent was placed. On day 71, the ureteral stent was retrieved.

The body weight of the pig fed 5% hydroxyproline was 30.80 kg at the beginning of the experiment (baseline) and 43.90 kg at the end of the experiment, with an increasing trend. The body weight of the pig fed 1% ethylene glycol and vitamin D was 28.55 kg at the beginning of the experiment (baseline) and slightly decreased to 26.32 kg at the end of the experiment (Fig. 3A). And, the animal doctors check their health status, daily feeding and water intake, and urine saturation levels every day and made sure it is safely done.

The number of crystals in the urine of the pig fed 5% hydroxyproline increased from 9.80 to 95.80 compared to the beginning and end of the experiment. The number of crystals in the urine of the pig fed 1% ethylene glycol and vitamin D increased from 7.00 to 101.60 compared to the beginning and end of the experiment (Fig. 3B).

Urinary Ca in the pig fed 5% hydroxyproline increased until day 28, reaching a maximum value of 69.13 (mM) on day 35, but showed a decreasing trend after day 45. Urinary Ca in the pig fed 1% ethylene glycol and vitamin D varied with load, with a maximum value of 79.50 (mM) at day 67. The mean urinary Ca during the stenting period was 37.83 mM for the pig fed 5% hydroxyproline, compared with 50.22 mM for the pig fed 1% ethylene glycol and vitamin D (Fig. 3C).

**Figure 4:**
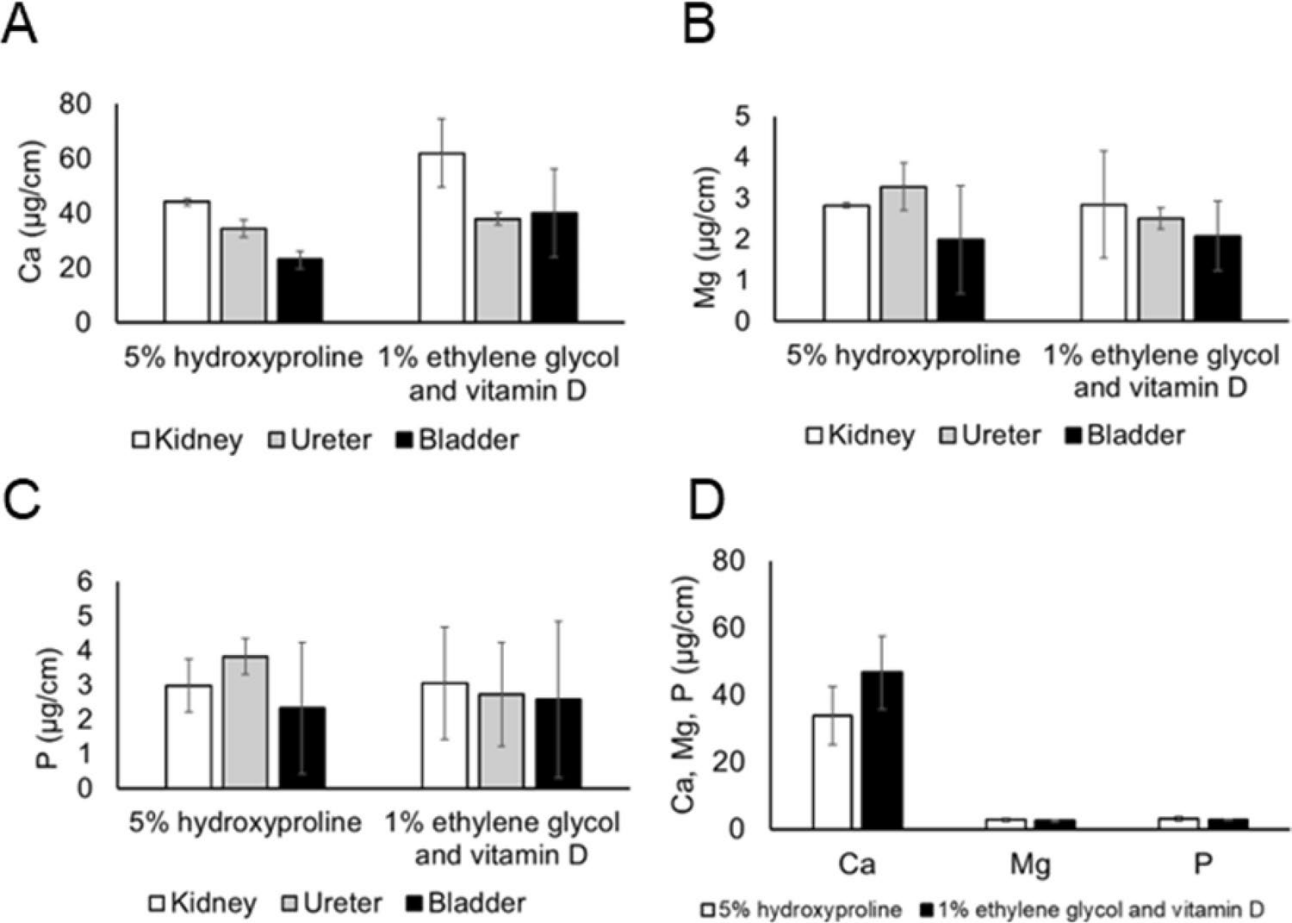
Quantitative analysis of crystal (Ca, Mg, P) adherence to the stents in a pig model. (**A**) (**B**) and (**C**) show quantitative analysis of crystal (Ca, Mg, P) for kidney, ureter, and bladder. (**D**) shows the average quantitative results (Ca, Mg, P) for kidney, ureter, and bladder.

ICP-OES analysis of the removed ureteral stent at three sites on the renal, urogenital and bladder sides showed that Ca deposition was higher on the kidney side than on the ureter side and bladder side. For Mg and P, there was no significant difference in the amount of deposition between sites (Fig. 4).

## Discussion

Ureteral stents, which are used to prevent urinary tract obstruction in urological patients, are prone to obstruction due to urinary crystal adhesion^1,2^. This study describes an artificial urine model and an animal model to evaluate the effectiveness of new ureteral stents designed to prevent stone adhesion to reduce stent-related adverse events such as pain, infection, bloody urine, and irritative bladder symptoms^21,22^. In the development of novel biomaterials for stents, rapid and reproducible in vitro testing procedures are desired to facilitate the evaluation of potential materials. In vitro models have been used primarily to assess stent encrustation, durability, and bacterial resistance. While these models provide valuable insight into the underlying mechanisms of biofilm formation and incrustation, they are far from reflecting the true state of affairs within the urinary tract. Many authors argue that static models using artificial urine do not mimic complex human urinary tract models^23^. However, Dynamic models such as the Modified Robbins Device (MRD) and the Center for Disease Control (CDC) biofilm reactor are essential for prototype testing, but not for screening testing. In addition, contamination is a problem for dynamic models. In addition, the cost of experiments is high^24,25^. We suggest that our experimental models are a static model that is quick and easy to use as a screening test to evaluate new stent materials.

Crystal adhesion to ureteral stents is related to pH, solubility and supersaturation. Crystal formation depends on solubility and supersaturation, and the relationship between supersaturation and crystal nucleation and growth is defined by the equation proposed by Nyvlt^26^. Increasing supersaturation increases crystal nucleation and growth. Artificial urine with a pH of 6.00 at the start of the experiment is weakly acidic, within the reference range of human urine pH, and contains ammonium chloride and urea in its composition^26^.

Ammonium chloride contains ammonia. These slightly precipitated ammonium ions increase the pH of the solution, causing calcium oxalate and calcium phosphate to become supersaturated and crystals to precipitate on the surface of the ureteral stent^27,28^. The solubility of calcium oxalate is 6.7 mg/ L. The solubility of calcium phosphate is 3.1 mg/ L^29^. The three types of artificial urine contain calcium chloride, sodium oxalate and sodium dihydrogen phosphate above the solubility level. However, comparing crystal adhesion in artificial urine, model 1 has higher concentrations of calcium chloride compared to artificial urine models 2 and 3. Also, since artificial urine model 3 is based on the average urine composition of a healthy person, it has a lower Ca concentration than model 1 and 2^10–12^. Pak et al. found that crystal growth of brushite increased with an increase in Ca concentration^10^, and indeed artificial urine model 1 had the most crystals attached to the ureteral stent during the 4-week period.

Evaluating the need for urea in the artificial urine model 1, the pH of artificial urine containing urea increased due to hydrolysis of urea contained in the artificial urine to produce carbon dioxide and ammonia. Also, Urea is isomeric to ammonium cyanate, producing a small amount of cyanide and ammonium ions in the artificial urine^30^. This causes a rapid increase in the pH of the artificial urine. Crystals grow from crystal nuclei on the surface of the stent, The urea-containing artificial urine had a strong pH increase, whereas the urea-free artificial urine, which had a slower pH increase, produced crystal nuclei on the surface of the ureteral stent as a starting point for crystal growth. Calcium oxalate crystals and calcium phosphate crystals account for the majority of renal stones^31,32^. We suggest that a gradual increase in the pH of the solution due to slightly precipitated ammonium ions leads to supersaturation of calcium oxalate crystals and calcium phosphate crystals and the precipitation of crystals on the surface of the ureteral stent^26^. However, If the pH and alkalinity of artificial urine containing urea increases too rapidly, the precipitation of magnesium ammonium magnesium phosphate crystals increases, conditions that are not suitable for calcium crystals precipitation^33^. Therefore, we suggest that conditions with a gradual increase in pH without urea are more suitable for calcium crystals formation than conditions with a rapid increase in pH, such as those containing urea.

When the starting pH of artificial urine was raised to pH 6.30, crystal formation seemed to be rapid and crystallization seemed to occur. When the pH was raised, the precipitation of calcium crystals accelerated, and the number of crystals adhering to the ureteral stent also increased. During the 5 week immersion, Ca on the ureteral stent immersed in artificial urine with a starting pH of pH 6.30 was also higher than in artificial urine with a starting pH of pH6.00, pH6.10 and pH6.20, indicating that crystal adherence to ureteral stents correlates with Ca.

As shown in the supplementary material, SEM–EDS analysis (C, O, Ca, P) suggested that calcium phosphate crystals were deposited. However, when combined with the fact that oxalic acid was found at 8.67 µg/cm, we consider that calcium oxalate as well as calcium phosphate was deposited simultaneously^34^.

In the rat experimental model, since the crystals found in urine on day 35 and on day 42 had the octahedral structure of calcium oxalate crystals^35^. This was because ethylene glycol was metabolized into hyperoxaluria, resulting in the formation of calcium oxalate crystals in the urine. However, ethylene glycol and some of its metabolites are nephrotoxic, so it was necessary to monitor body weight during the experiment^14,19^. Jarald et al. used 0.75% ethylene glycol and induced kidney stones in 28 days in their study, but this study used a lower concentration of 0.4% ethylene glycol and was able to induce kidney stones in 35 days^14^. Renal stones were induced even at low concentrations of ethylene glycol because the additional administration of calcium chloride promoted hypercalciuria and the deposition of calcium oxalate crystals^36^.

The pigs did not increase exponentially with respect to their weight, but we suggest this was due to the fact that they were fed a high fiber diet that did not affect the study design. Soria F et al. suggest that ideally, interventions should be carried out on about 30 kg models, as the dimensions of their urinary tract at that weight are comparable to a human adult^15,16^.

The increase in urinary crystals in the pig fed 5% hydroxyproline was due to hyperoxaluria, as hydroxyproline, a precursor of oxalic acid, was metabolized to oxalic acid, and Ca in the urine promoted calcium oxalate crystals^17–20^. The increase in urinary crystals in the pig fed 1% ethylene glycol and vitamin D was also due to hyperoxaluria, as ethylene glycol was metabolized to oxalic acid, and the additional administration of vitamin D promoted calcium oxalate crystals by increasing the rate of Ca absorption in the body. The fact that the maximum urinary Ca value was higher in the pig with 1% ethylene glycol and vitamin D also indicates that vitamin D promoted Ca absorption in the body^14,28^. The lack of correlation between urinary crystals and urinary Ca concentrations is due to the fact that when crystals were counted under a microscope, they were counted regardless of size, and because of weak magnification, magnesium ammonium phosphate crystals and other urinary crystals were counted.

The fact that Ca deposition was greater on the renal side than on the ureteral or bladder side is reasonable because most clinically important stones grow in the renal papillae^34,37,38^.

A model of crystal deposition in urine could be established for both hydroxyproline and ethylene glycol + vitamin D. Ethylene glycol + vitamin D may be more likely to increase urinary Ca concentrations and precipitate calcium oxalate crystals. However, ethylene glycol and some of its metabolites are nephrotoxic and body weight should probably be monitored during the experiment.

Artificial urine of several compositions is sensitive to pH, temperature and environment. Also, the crystals observed under the microscope were crystals adhering to the outside of the ureteral stent, and not all adhering crystals could be observed, as crystals normally adhere to the inside of the ureteral stent. However, the results correlated with the quantitative results for Ca on the ureteral stent in vitro, which is a useful comparison. The in vivo experiment was also valuable because it allowed the potabilization of a model in which crystals in the urine adhered to the stent surface in a short period of time, although the n was small. In addition, we have not tested the urine culture, and had no control group. In the future, the number of animal models will be increased and urine samples will be analyzed in more detail (in vivo pH, urinary leukocyte count, urinary bacterial count, presence of infection, etc.) with the check of urine culture and set of control group.

In conclusion, in vitro crystal adhesion to the surface of ureteral stents can be expected when the ureteral stent is immersed for a period of 5 weeks at an initial pH of 6.30 using the urea-free artificial urine model 1. In vivo, in the rat experimental model crystals were found in the urine after 35 days when 0.4% ethylene glycol and 1% ammonium chloride was administered. In the pig experimental model, urinary crystals increased when either 5% hydroxyproline or 1% ethylene glycol + vitamin D was administered, but urinary Ca was more likely to increase in the model with 1% ethylene glycol + vitamin D. This in vitro and in vivo experimental model can be used to evaluate the effectiveness of newly developed ureteral stents for preventing crystal adhesion, as part of the development of improved ureteral stents.

## Supplementary Information

Supplementary Information.

## Data Availability

The datasets used and/or analyzed during the current study available from the corresponding author on reasonable request.
