# Author Correction: Establishment of an artificial urine model in vitro and rat or pig model in vivo to evaluate urinary crystal adherence

**DOI:** 10.1038/s41598-025-31907-0

**Published:** 2025-12-18

**Authors:** Kana Hayashi, Katsumi Shigemura, Hiroshi Tanimoto, Kazuo Kumagai, Ralph Rolly Gonzales, Young-Min Yang, Koki Maeda, Hideto Matsuyama, Masato Fujisawa

**Affiliations:** 1 Division of Infectious Diseases, Department of Public Health, Kobe University Graduate School of Health Sciences, 7-10-2 Tomogaoka Suma-Ku, Kobe, 654-0142 Japan; 2 Department of Urology, Teikyo University Graduate School of Medicine, 2-11-1 Kaga, Itabashi-Ku, Tokyo, 173-8605 Japan; 3 Research Center for Membrane and Film Technology, Kobe University, 1-1 Rokkodaicho, Nada-Ku, Kobe, 657-8501 Japan; 4 Department of Chemical Science and Engineering, Kobe University, 1-1 Rokkodaicho, Nada-Ku, Kobe, 657-8501 Japan; 5 Division of Urology, Kobe University Graduate School of Medicine, 7-5-1 Kusunoki-Cho, Chuo-Ku, Kobe, 650-0017 Japan

Correction to: *Scientific Reports* 10.1038/s41598-024-62766-w, published online 25 May 2024

The Funding section in the original version of this Article was incomplete.

“This study was supported by the Uehara Memorial Foundation, a Grant-in-Aid for Research Activity Start-up (20K22979) from the Japan Society for the Promotion of Science (JSPS) and Suzuken Memorial Foundation (20-014).”

now reads,

“This study was supported by the Uehara Memorial Foundation, a Grant-in-Aid for Research Activity Start-up (20K22979) from the Japan Society for the Promotion of Science (JSPS) and Suzuken Memorial Foundation (20-014), and AMED (JP23ym0126805, A222).”

The original Article has been corrected.

