# Supplementary material for: Establishment of an artificial urine model in vitro and rat or pig model in vivo to evaluate urinary crystal adherence
Source: Sci Rep. 2024 May 25;14:12001. doi: 10.1038/s41598-024-62766-w (PMC11127959; doi:10.1038/s41598-024-62766-w)
Supplement: Supplementary file 1 — Supplementary Information. [file 41598_2024_62766_MOESM1_ESM.docx]

**Establishment of an artificial urine model *in vitro* and rat or pig model *in vivo* to evaluate urinary crystal adherence**

Kana Hayashi^1^, KatsumiShigemura^2^, HiroshiTanimoto^1^, KazuoKumagai^3 4^, Ralph RollyGonzales^3^, Young-MinYang^5^, Koki Maeda^5^, Hideto Matsuyama^3 4^ , MasatoFujisawa^5^

^1^Division of Infectious Diseases, Department of Public HealthKobe University Graduate School of Health Sciences7-10-2 Tomogaoka, Suma-Ku, Kobe 654-0142 Japan

^2^ Department of Urology, Teikyo University Graduate School of Medicine 2-11-1 Kaga, Itabashi-Ku, Tokyo 173-8605 Japan

^3^ Research Center for Membrane and Film Technology, Kobe University1-1 Rokkodaicho, Nada-Ku, Kobe 657-8501 Japan

^4^ Department of Chemical Science and Engineering, Kobe University1-1 Rokkodaicho, Nada-Ku, Kobe 657-8501 Japan

^5^ Division of Urology, Kobe University Graduate School of Medicine7-5-1 Kusunoki-Cho, Chuo-Ku, Kobe 650-0017 Japan

Corresponding Author:

Katsumi Shigemura,

Department of Urology, Teikyo University Graduate School of Medicine2-11-1 Kaga, Itabashi-Ku, Tokyo 173-8605, Japan TEL +81-3-3964-1211 FAX +81-3-3964-8934

e-mail: [shigemura.katsumi.up@teikyo-u.ac.jp](mailto:shigemura.katsumi.up@teikyo-u.ac.jp)

**Supplementary Fig. S1: The chart illustrating our experimental artificial urine model.**


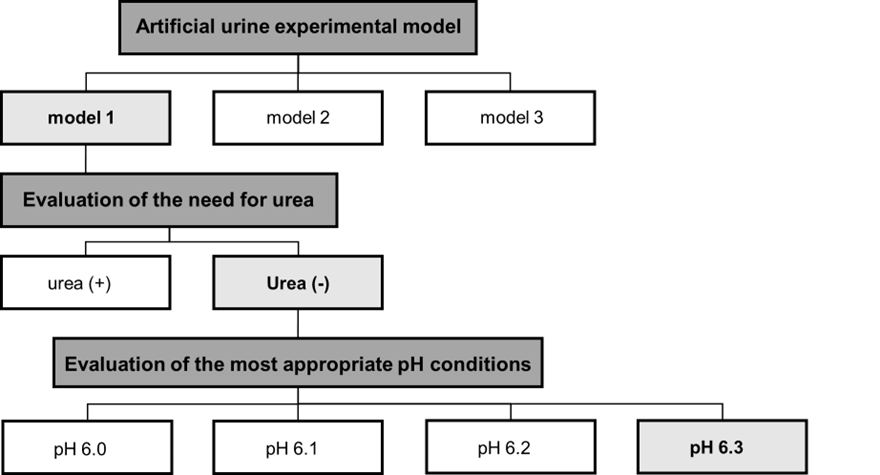


Supplementary Fig. S1 represents the procedure of the artificial urine experimental model, in which experiments were conducted in the order “Artificial urine experimental model”, “Evaluation of the need for urea”, and “Evaluation of the most appropriate pH conditions”.

**Supplementary Fig. S2: Surface morphology and elemental mapping of stents using SEM.**


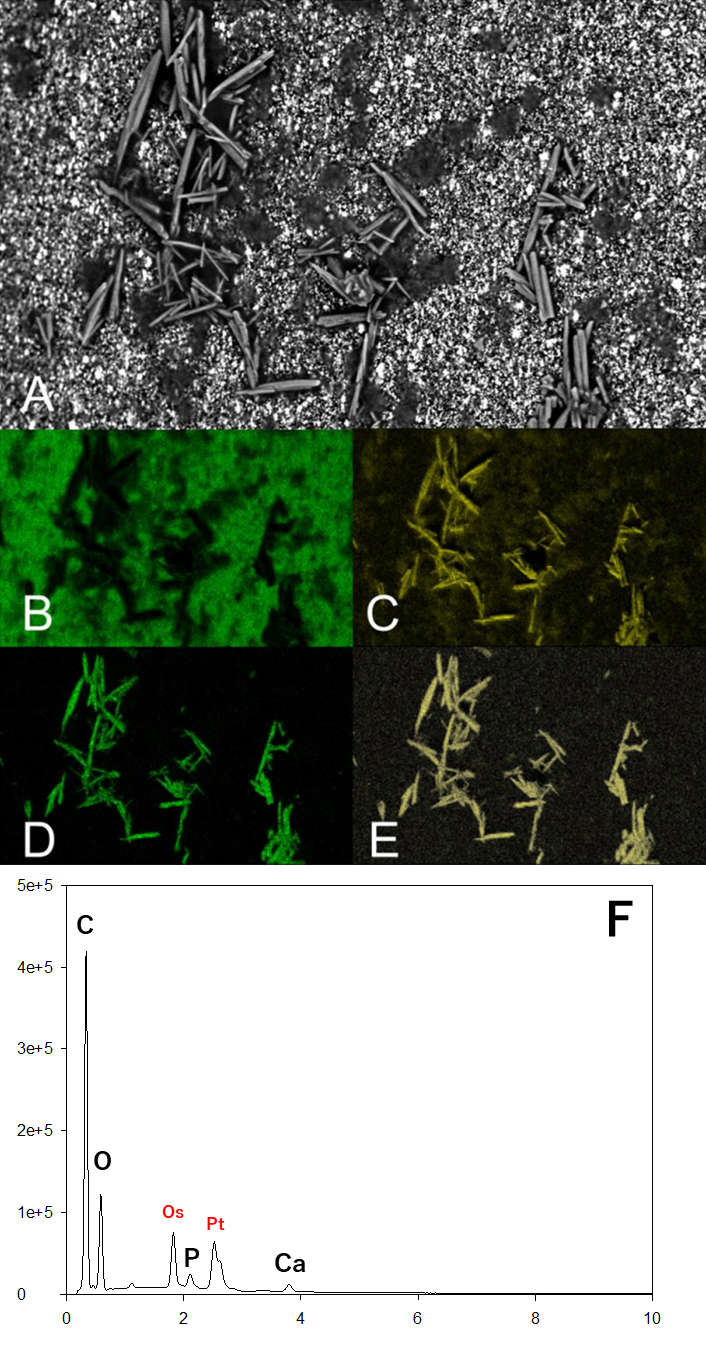


(A) shows stent surface morphology in the artificial urine experiment.

(B), (C), (D) and (E) show elemental mapping of stents in the artificial urine experiment, in which (B), (C), (D) and (E) show C, O, Ca and P, respectively.

(F) shows SEM-EDS elemental point spectrum.
